# A phase transition caught in mid-course: independent and concomitant analyses of the monoclinic and triclinic structures of (^*n*^Bu_4_N)[Co(orotate)_2_(bipy)]·3H_2_O

**DOI:** 10.1107/S2053229617010841

**Published:** 2017-08-17

**Authors:** Miguel Castro, Larry R. Falvello, Elena Forcén-Vázquez, Pablo Guerra, Nuha A. Al-Kenany, Gema Martínez, Milagros Tomás

**Affiliations:** a Departamento de Ciencia y Tecnología de Materiales y Fluidos, Escuela de Ingeniería y Arquitectura, Instituto de Ciencia de Materiales de Aragón (ICMA), University of Zaragoza–CSIC, María de Luna 3, Zaragoza E-50018, Spain; b Department of Inorganic Chemistry and Aragón Materials Science Institute (ICMA), University of Zaragoza-CSIC, Pedro Cerbuna 12, Zaragoza E-50009, Spain; c Department of Inorganic Chemistry and Instituto de Síntesis Química y Catálisis Homogenea (ISQCH), University of Zaragoza–CSIC, Pedro Cerbuna 12, Zaragoza E-50009, Spain

**Keywords:** order–disorder phase transition, crystal structure, single-crystal-to-single-crystal transformation, tetra­butyl­ammonium disorder, orotate, cobalt, vitamin B_13_ complex

## Abstract

The preparation and characterization of the ^*n*^Bu_4_N^+^ salts of two bis-orotate(2−) complexes of cobalt, namely bis­(tetra-*n*-butyl­ammonium) di­aqua­bis­(2,4-dioxo-1,2,3,4-tetra­hydro­pyrimidin-1-ide-6-carboxyl­ato-κ^2^ *N* ^1^,*O* ^6^)cobalt(II) 1.8-hydrate, (C_16_H_36_N)_2_[Co(C_5_H_2_N_2_O_4_)_2_(H_2_O)_2_]·1.8H_2_O, (**1**), and tetra-*n*-butyl­ammonium (2,2′-bi­pyridine-κ^2^ *N*,*N*′)bis­(2,4-dioxo-1,2,3,4-tetra­hydro­pyrimidin-1-ide-6-carbox­yl­ato-κ^2^ *N* ^1^,*O* ^6^)cobalt(III) trihydrate, (C_16_H_36_N)[Co(C_5_H_2_N_2_O_4_)_2_(C_10_H_8_N_2_)]·3H_2_O, (**2**), are reported. The Co^III^ complex, (**2**), which is monoclinic at room tem­perature, presents a conservative single-crystal-to-single-crystal phase transition below 200 K, producing a triclinic twin. The transition, which involves a conformational change in one of the ^*n*^Bu groups of the cation, is reversible and can be cycled. Both end phases have been characterized structurally and the system was also characterized structurally in a two-phase inter­mediate state, using single-crystal diffraction techniques, with both the monoclinic and triclinic phases present. Thermal analysis allows a rough estimate of the small energy content, *viz.* 0.25 kJ mol^−1^, for both the monoclinic-to-triclinic transformation and the reverse transition, in agreement with the nature of the structural changes involving only the ^*n*^Bu_4_N^+^ cation.

## Abstract 2

Repeated cycling through a reversible order–disorder phase transition in a crystal of a cobalt orotate complex produces an arrested transformation, leaving the sample with both triclinic and monoclinic domains. Independent determinations of the two structures were carried out from single-phase samples and the partial transition permitted simultaneous structure determination of the two phases at a temperature at which one of them is not expected to exist independently.

## Introduction   

We have prepared the ^*n*^Bu_4_N^+^ salt of one isomer of the simple transition metal complex [Co(Or)_2_(bipy)]^−^ [Or^2−^ is oro­tate(2−) (see Scheme 1) and bipy is 2,2′-bipyridyl] and have observed that at a temperature near 180 K it undergoes a phase transformation for which the two components can be analyzed structurally at the same time using single-crystal diffraction techniques.
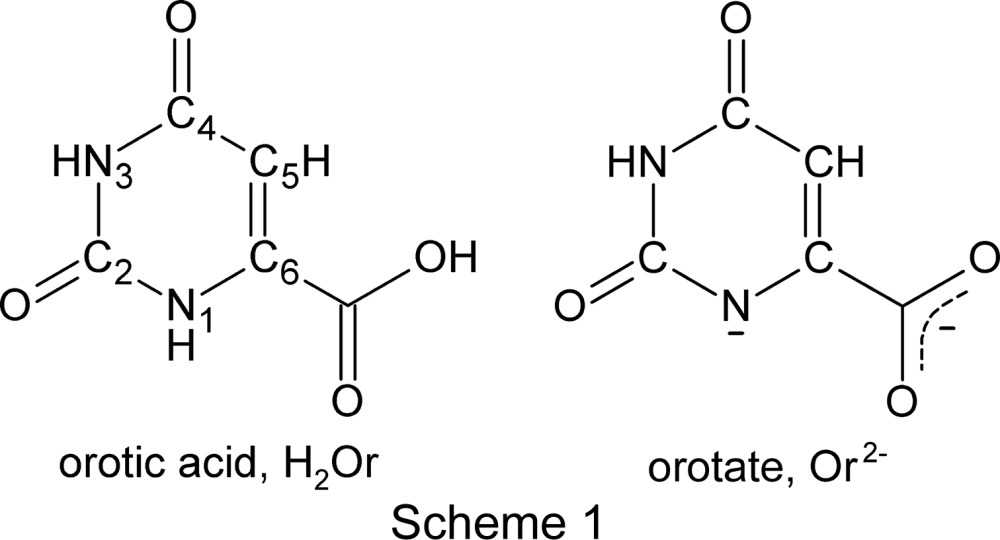


Orotate(2−) is the dianion of orotic acid (H_2_Or or 2,6-dioxo-1,2,3,6-tetra­hydro­pyrimidine-4-carb­oxy­lic acid), known as vitamin B_13_ (although it is understood not to be a vitamin), a biologically important mol­ecule that is the precursor for the pyrimidine bases in living systems and which is important in other biological processes (Loeffler *et al.*, 2016 ▸). Orotate has been used in the preparation of a stable salt of tenofovir disoproxil, an anti­viral agent used against the HIV and hepatitis B viruses (Park *et al.*, 2014 ▸). Our own inter­est in orotic acid and its salts arises from the five varied functional groups that gird its periphery, which make it a versatile ligand in transition-metal chemistry. It is capable of coordinating to a transition-metal atom in different ways and at the same time of participating in significant directional noncovalent inter­actions with its environment, including crystalline environments. We have referred to such chemical entities as ‘polyfunctional ligands,’ a name that reflects the presence of numerous functional groups rather than any putative mechanical or physical functionality.

Orotate complexes have been studied structurally in detail; at the time of writing, some 131 crystal structures of orotate complexes of transition metals have been recorded in the Cambridge Structural Database (CSD; Groom *et al.*, 2016 ▸), along with 15 complexes of lanthanoid elements and a single complex with a heavy rare earth element, *i.e.* uranium (Mentzafos *et al.*, 1987 ▸). In these complexes, orotate is usually found to be doubly deprotonated, at the carboxyl­ate group and at atom N1. By far the commonest coordination mode observed for orotate is chelation through the N1 atom and one of the carboxyl­ate O atoms.

In a separate line of development, we note that mol­ecular solids containing the *n*-butyl­ammonium fragment have been observed to undergo order–disorder phase transitions involving changes in the conformation of as little as one butyl arm of the cation (Willett *et al.*, 2005 ▸). Inter­est in the preparation of mol­ecular materials that undergo phase transitions arises from the possibility of switching chemical and physical properties, such as spectroscopic characteristics (Falvello *et al.*, 1999 ▸), magnetic and electric properties, and others, in a controllable manner (Fujita & Awaga, 1999 ▸; Sato *et al.*, 2007 ▸; Schneemann *et al.*, 2014 ▸; Li *et al.*, 2016 ▸; Paglione & Greene, 2010 ▸; MacFarlane & Forsyth, 2001 ▸; Mason *et al.*, 2015 ▸; Nauha *et al.*, 2016 ▸; Rodríguez-Velamazán *et al.*, 2012 ▸). This phenomenon has been observed in materials of potential technological importance (Pielichowska & Pielichowski, 2014 ▸; Szaciłowski, 2008 ▸). Particularly inter­esting are single-crystal-to-single-crystal transformations (SCSC), which provide valuable information for the understanding of the switching of the properties of those mol­ecular materials, since both the mother and daughter phases can be structurally characterized.

One way to obtain solids that can undergo phase transitions while maintaining their crystallinity is by using mol­ecular fragments for which there exist potential structural changes requiring low energy and demanding little difference between the sizes and shapes of the initial and final species. Straight-chain paraffins have long been recognized as satisfying these criteria (Müller, 1932 ▸). Indeed, rotator and/or plastic phase transitions have been observed for crystals with *n*-alkyl­ammonium salts with small anions, such as halides (Shimizu *et al.*, 1997 ▸), mainly through characterization by thermal analysis and nuclear magnetic resonance (NMR) techniques. Re­gar­ding the *n*-butyl group in particular, ^*n*^Bu_4_NI, a simple salt, presents both a phase transition and ionic transport (Asayama *et al.*, 2005 ▸, 2006 ▸); however, very few di-*n*-butyl (57 structures in the CSD, only two phase transitions; Peng *et al.*, 2008 ▸; Khan *et al.*, 2015 ▸) and tri-*n*-butyl­ammonium compounds (49 structures in the CSD, two phase transitions) have been involved in phase transitions which have been characterized by single-crystal X-ray diffraction (Asghar *et al.*, 2015 ▸, 2016 ▸).

In contrast to di- and tri-*n*-butyl­ammonium, tetra-*n*-butyl­ammonium is a more widely used cation, especially as a counter-ion for coordination compounds; there are more than 80 times as many structures with ^*n*^Bu_4_N^+^ as with ^*n*^Bu_3_NH^+^ or ^*n*^Bu_2_NH_2_^+^ (4742/49/57 entries in the CSD, respectively). Yet, and in spite of the high percentage of crystal structures with this group in disorder, the number of phase transitions explicitly characterized by X-ray diffraction has also been very low for systems involving tetra-*n*-butyl­ammonium (Czerwonka *et al.*, 1988 ▸; Excoffon *et al.*, 1991 ▸; Watase *et al.*, 2003 ▸; Willett *et al.*, 2005 ▸).
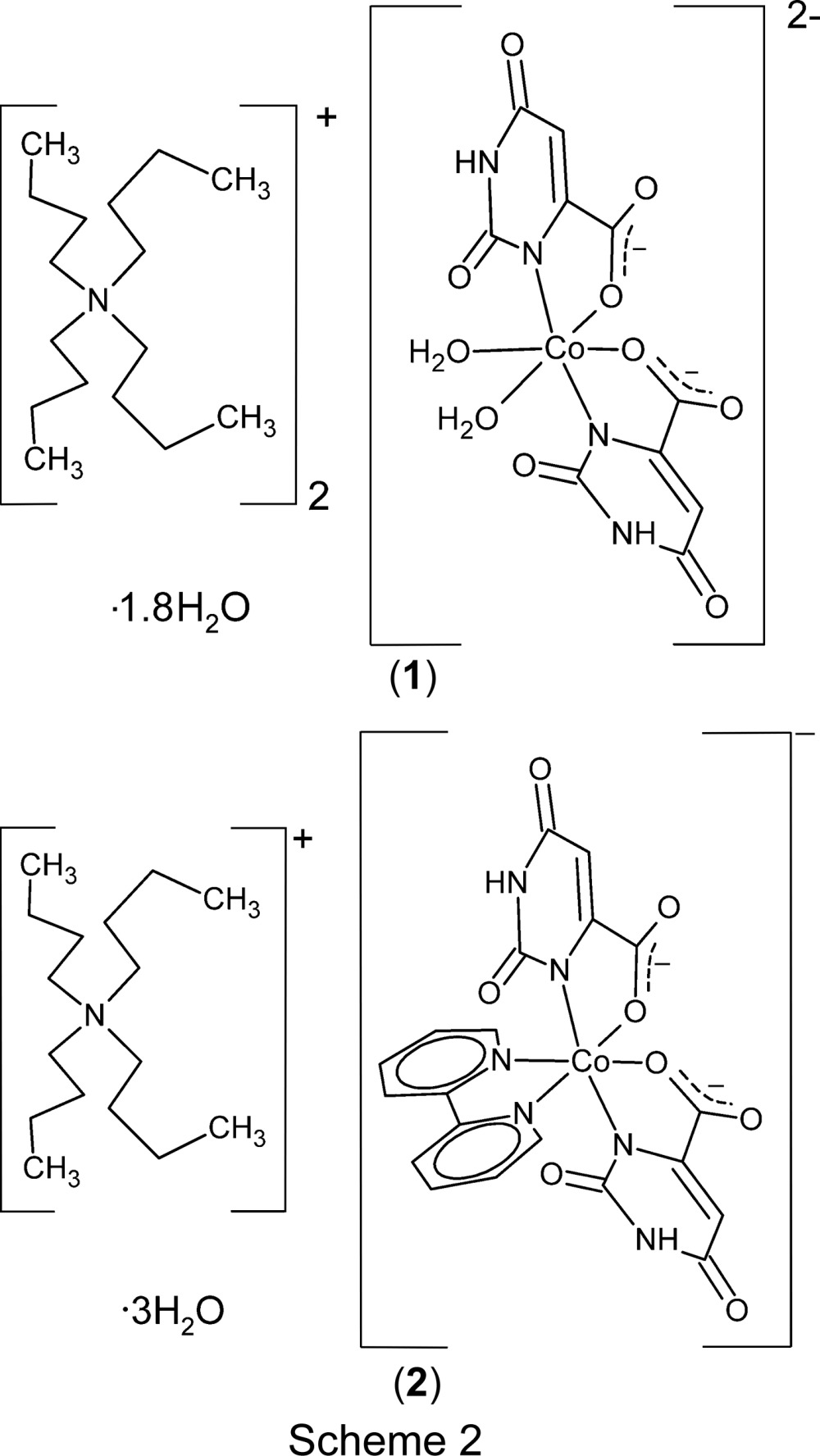


The term ‘partial phase transition’ has been used for what are now a large number of systems concluded to have undergone phase transitions in part of the volume of a substance and not in the rest. Most, by far, of the systems observed to behave in such a way have been inorganic solids. Recent examples in which partial phase transitions have been imputed include that of a hydrogen-storage material (Luo *et al.*, 2016 ▸) and a partial transition observed in a lithium-containing spinel, *i.e.* LiMnTiO_4_, a solid with potential relevance as a cathode material for rechargeable lithium-ion batteries (Murphy *et al.*, 2015 ▸). As for molecular solids, a partial phase transition was proposed for the α and β polymorphs of dl-norleucine, based on mol­ecular dynamics simulations at temperatures for which the phases are stable and metastable (van den Ende & Cuppen, 2014 ▸). We are not aware of the full structural characterization of an arrested phase transition in a mol­ecular crystal.

In what follows, we report the preparations of (^*n*^Bu_4_N)_2_[Co(Or)_2_(H_2_O)_2_]·1.8H_2_O, (**1**), and (^*n*^Bu_4_N)[Co(Or)_2_(bipy)]·3H_2_O, (**2**), the hydrated tetra­butyl­ammonium salts of simple Co^II^ and Co^III^ coordination complexes. For (**2**), we report its phase transition from a room-temperature dynamically disordered monoclinic phase to a low-temperature ordered but twinned triclinic phase. Upon cycling, this phase transition was observed to halt with part of the sample in each phase. This double-phase sample was characterized structurally using single-crystal X-ray diffraction techniques based on in-house measurements, and the single composite diffraction pattern yielded two high-quality structure analyses. In addition to permitting accurate characterization of both phases, the analysis of the two-phase sample using nominally single-crystal techniques permitted the characterization of the monoclinic phase at a temperature at which in principle it would not normally exist.

## Experimental   

### General   

All reagents were used as received without further purification. The IR spectra of compounds (**1**) and (**2**) were recorded in the 4000–300 cm^−1^ range on a PerkinElmer Spectrum 100 FT–IR spectrophotometer equipped with an ATR accessory. Elemental analyses were performed on a PerkinElmer 240 Series II microanalyzer.

### Syntheses   

#### Preparation of (^*n*^Bu_4_N)_2_[*cis*-Co(Or)_2_(H_2_O)_2_]·*x*H_2_O, (1)   

A mixture of CoCO_3_·H_2_O (0.50 g, 3.65 mmol), orotic acid hydrate (C_5_H_4_O_4_N_2_·H_2_O; 1.27 g, 7.30 mmol) and water (50 ml) was stirred for 2 h in air allowing gas evolution, then a solution of ^*n*^Bu_4_NOH (1.5 *M*, 40%) in H_2_O (7.15 mmol, 4.76 ml) was added. The resulting suspension was filtered and left standing for evaporation. Orange crystals of (**1**) were obtained from the filtrate after several days in 75% yield (2.48 g, 2.59 mmol). Analysis calculated (%) for (^*n*^Bu_4_N)_2_[*cis*-Co(Or)_2_(H_2_O)_2_]·1H_2_O – *i.e.* one mol­ecule of unligated H_2_O per formula unit – C_42_H_82_CoN_6_O_11_: C 55.67, H 9.12, N 9.28; found: C 55.79, H 8.97, N 9.18. IR (cm^−1^): 2960 (*s*), 2876 (*m*), 1643 (*s*), 1585 (*s*), 1563 (*s*), 1464 (*s*), 1361 (*s*), 1014 (*m*), 878 (*m*), 786 (*s*). **Note**: the crystal structure determination produced a model with 1.8 free H_2_O units per formula unit of the complex for the crystal from which the diffraction data were measured.

#### Preparation of (^*n*^Bu_4_N)[Co(Or)_2_(bipy)]·3H_2_O, (2)   

A mixture of orotic acid hydrate (C_5_H_4_O_4_N_2_·H_2_O; 5.855 g, 33.63 mmol), CoCO_3_·H_2_O (2 g, 14.60 mmol) and water (190 ml) was stirred for 2 h at 333 K. The flask was evacuated periodically by means of a water pump and then left stirring overnight. After that time, the flask was evacuated once more and then a solution of ^*n*^Bu_4_NOH (11 ml, 16.81 mmol, 1.53 *M*, 40%) was added. The resulting suspension was stirred at room temperature for 1 h. 2,2′-Bi­pyridine (2.6261 g, 16.815 mmol) was added and, after 15 min, H_2_O_2_ (2.06 ml, 20.2 mmol, 30%, 9.79 *M*) was added. The colour of the mixture turned to deep wine red and the suspension was filtered. Red crystals of (**2**) were obtained from the solution after 15 h in 40% yield (4.8376 g, 5.91 mmol). Posterior evaporation of the remaining solution produced more of compound (**2**), but mixed with other cobalt orotate compounds. Analysis calculated (%) for C_36_H_54_CoN_7_O_11_, *cis*-(**2**): C 52.74, H 6.64, N 11.96; found: C 52.95, H 6.68, N 12.19. IR (cm^−1^): 3388 (*m*), 2964 (*m*), 2788 (*m*), 1642 (*s*), 1610 (*s*), 1462 (*s*), 1398 (*s*), 1351 (*s*), 1294 (*s*), 1151 (*m*), 1027 (*m*), 948 (*m*), 882 (*m*), 767 (*s*), 594 (*m*), 454 (*s*), 418 (*s*).

### Thermal analysis measurements   

Thermal analysis measurements were performed using a differential scanning calorimeter (DSC) Q1000 from TA Instruments equipped with a liquid-nitro­gen cooling system, allowing temperatures to reach 100 K. A powder sample of approximately 10 mg mass was sealed in a nonhermetic flat aluminium capsule. Thermograms, both on heating and cooling, were performed at a scan rate of 10 K min^−1^. Temperature and enthalpy calibrations were made with an indium standard sample by using its melting data. Comparison with expected values shows very small changes in the onset temperature (<0.1 K) and in the enthalpy content (<1.5%). In order to determine the heat-capacity anomalies and their enthalpy contents, a smooth baseline, obtained by fitting the thermograms outside of the transition temperature range with a linear or low-degree polynomial function, was subtracted from the thermogram. In the present case, anomalies are small and diffuse, and this procedure, using a more or less arbitrary baseline, increases significantly the uncertainty in the enthalpy determination; thus, the reported values must be considered as rough estimates.

### Single-crystal X-ray structure determination of compounds (1) and (2)   

Single-crystal diffraction data were measured using Oxford Diffraction Xcalibur S3 four-circle diffractometers equipped with graphite-monochomated Mo *K*α radiation (λ = 0.71073 Å). Oxford Instruments CryoJetLT and CryoJetHT nitro­gen-flow temperature controllers were used to maintain the samples of compound (**2**) at set temperatures. The samples were mounted on Mitegen supports and covered with Fomblin oil. Multiscan absorption correction procedures were applied to the data and used to derive error models (Blessing, 1995 ▸, 1997 ▸). The crystallographic parameters and refinement residuals for all of the structure analyses are given in Tables 1 ▸ and 2 ▸.

#### (^*n*^Bu_4_N)_2_[*cis*-Co(Or)_2_(H_2_O)_2_]·*x*H_2_O, (1)   

The crystal structure determination of (**1**) at room temperature produced a structural model with 1.8 units of unligated water per formula unit. Structure solution and refinement were routine except for two disorder assemblies whose atomic sites were located in Fourier maps and refined with partial occupancies for the respective disorder groups. The first disorder assembly involved a γ-methyl­ene group (C23) of one of the ^*n*^Bu_4_N^+^ cations. Two sites were located for the C atom and their occupancies were initially refined with a constraint to a total population of 1.0. The occupancies refined to values close to 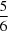 and 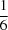, and so were fixed at these values for the final refinement. H atoms for the disordered congeners were placed at calculated positions and refined as riding atoms, with displacement-parameter constraints. The partially occupied H-atom sites included those of the adjacent CH_2_ and CH_3_ groups at atoms C22 and C24. For the latter, the H-atom coordinates were calculated so as to have staggered conformations with respect to atom C23. A second disorder assembly was found for the inter­stitial O4*W* water site. The populations of the two sites were initially refined with a constraint to a sum of 1.0. The resulting population parameter converged to a value of 0.798 (5) for O4*WA*, and so the site-occupancy factors were fixed at 0.8 (O4*WA*) and 0.2 (O4*WB*) for the final refinement. Since the remaining inter­stitial water site, at O3*W*, makes an impossibly short contact with an inversion-related congener of O4*WB*, O3*W* was treated as a member of the disorder assembly and also had a fixed population of 0.8 in the final refinement. The H atoms attached to O3*W*, O4*WA* and O4*WB* were not located. All of the H atoms of the ^*n*^Bu_4_N^+^ cations were placed at calculated positions based on geometry for CH_2_ and on local slant Fourier calculations for the CH_3_ groups not affected by disorder. *U*_iso_(H) values for methyl­ene and methyl H atoms were constrained to 1.2 and 1.5 times the *U*_eq_ values of their respective carrier C atoms. H atoms of the orotate groups and ligated water molecules were located in difference Fourier maps and refined with independent coordinates and with isotropic displacement parameters constrained to 1.2*U*_eq_ of the carrier atom for the orotate H atoms and with H-atom *U*_iso_ values freely refined for the aqua ligands.

#### (^*n*^Bu_4_N)[Co(Or)_2_(bipy)]·3H_2_O, (2)   

We report five structure analyses for compound (**2**). The structure of the crystals as prepared is monoclinic, space group *P*2/*n*, analyzed at *T* = 277 K. For this analysis, *i.e.* (**2a**), all non-water H atoms were placed at calculated positions and refined as riders, with *U*_iso_ values set at 1.2 (nonmeth­yl) or 1.5 (meth­yl) times the *U*_eq_ values of the respective parent atoms. Water H atoms were located in a difference map and refined freely. The atoms of both of the independent ^*n*^Bu groups of the ^*n*^Bu_4_N^+^ cation showed increasing displacements on going from the α- to the β-C atoms, but the terminal –CH_2_CH_3_ group at atoms C22 and C23 showed quite pronounced transverse displacement accompanied by the shortened ‘apparent’ bond length of 1.182 (6) Å, normally attributed to libration.

When the same crystal is cooled to *T* = 100 K, it undergoes a transition to a triclinic phase (space group *P* 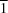), with twinning. The initial monoclinic phase, (**2a**) (Table 2 ▸), was analyzed using routine single-crystal X-ray procedures. The triclinic phase at *T* = 100 K, (**2b**), was treated as a ‘nonmerohedral twin’ (Herbst-Irmer, 2016 ▸) and the structure was refined using a combined data set (*SHELXL2014* ‘HKLF 5’) with the residuals given in Table 2 ▸. The twin ratios were calculated as 0.533 and 0.467 by the data integration program, and the transformation, in terms of cell-axis vectors, from the first to the second component, is:
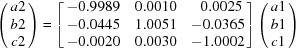
The unit cell and setting used for triclinic structure (**2b**) were chosen to correspond as closely as possible to the unit cell and setting of the initial monoclinic phase (**2a**). As a result, the triclinic cell is not the conventional reduced cell that would have been used if the structure of (**2b**) had been done independently of its monoclinic relative. The standard unit cell is *a* = 9.3791 (8), *b* = 12.9054 (8), *c* = 16.1290 (12) Å, α = 102.898 (6), β = 91.276 (6), γ = 91.472 (6)° and *V* = 1901.6 (2) Å^3^. The transformation from the unit cell used to the standard reduced cell and setting, in terms of unit-cell basis vectors, is the following, in which the primed axes are those of the conventional cell:
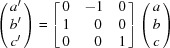
 Following the thermal analysis, which suggested that the monoclinic-to-triclinic transition occurs in the 160–220 K temperature range, we explored the diffraction pattern of a second crystal of (**2**) in the same range, beginning at the higher temperature. Firstly, the unit cell was determined at *T* = 220 K, confirming the exclusive presence of the monoclinic phase. The temperature was then cycled to *T* = 170 K and back in increments of 10 or 20 K, with an axial photo of [010] being taken at each temperature. Photos were made, in this order, for *T* = 220, 200, 190, 180, 170, 190, 200 and 220 K, and were taken after a 15 min inter­val at each temperature, except for the final *T* = 220 K, for which photos were taken after 20, 70 and 120 min. The photos showed progressive spot splitting as the temperature was lowered, and eventually showed the loss of mirror symmetry perpendicular to this axis. When the temperature was raised, the splitting progressively disappeared, with the axial photo returning to nearly its original appearance when the temperature had once again reached *T* = 220 K.

At this point, a full structure analysis was conducted at *T* = 220 K, *i.e.* (**2c**) (Table 2 ▸); this confirmed that the structure at this point was identical to the original monoclinic structure. The H atoms of the two independent unligated water sites were located in a difference Fourier map, and their positional and isotropic displacement parameters were refined freely.

The temperature was then lowered to *T* = 170 K, and a redundant sphere of data was gathered. The diffraction pattern revealed the presence of both the monoclinic and the triclinic phases. Because of the high redundancy, it was possible to isolate nearly complete data sets with reflections unique to each of the phases. Refinements were conducted routinely for both [*i.e.* monoclinic (**2d**) and triclinic (**2e**)]. For monoclinic (**2d**), the ^*n*^Bu group C20—C21—C22—C23 was found to have its terminal ethyl fragment disordered two ways, with the majority component (75%) having an *anti* conformation, as in the higher-temperature monoclinic structures, and with the minor component in a *syn* conformation, as in one of the ^*n*^Bu groups of the triclinic structure. Similarity restraints were applied to the Cγ—Cδ distances and to the 1,3-Cβ⋯Cδ distances. Similarity restraints were also used for the anisotropic displacement parameters of the Cγ and Cδ atoms of the disordered congeners. As was described above for triclinic structure (**2b**), the unit cell that was used for (**2e**) was chosen to correspond as closely as possible to that of the monoclinic structure. The transformation from the triclinic cell used to the conventional reduced-cell setting is the same as that given for (**2b**), and in the case of (**2e**) gives the conventional cell *a* = 9.4028 (14), *b* = 13.0155 (15), *c* = 16.2640 (17) Å, α = 103.054 (9), β = 91.206 (11), γ = 91.313 (11)° and *V* = 1937.8 (4) Å^3^.

## Results and discussion   

CoCO_3_·H_2_O reacts with orotic acid monohydrate, C_5_H_4_N_2_O_4_·H_2_O, and ^*n*^Bu_4_NOH in water at room temperature, giving different products depending on the reaction conditions. When the reaction was carried out in water with Co:H_2_Or:^*n*^Bu_4_NOH proportions of 1:2:2, the anionic Co^II^ derivative (^*n*^Bu_4_N)_2_[*cis*-Co(Or)_2_(H_2_O)_2_]·2H_2_O, (**1**), was formed (Equation 1).

CoCO_3_ + 2H_2_Or + 2NBu_4_OH 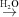 (NBu_4_)_2_[*cis*-Co(Or)_2_(H_2_O)_2_] + CO_2_↑ (1)

The same reaction for Co:H_2_Or:^*n*^Bu_4_NOH ratios of 1:2:1 in the presence of 2,2′-bi­pyridine (bipy) and H_2_O_2_ (Equation 2)

CoCO_3_ + 2H_2_Or + NBu_4_OH + bipy 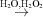 (NBu_4_)[*cis*-Co(Or)_2_(bipy)] + CO_2_↑ (2)

leads to the formation of the Co^III^ salt (^*n*^Bu_4_N)[*cis*-Co(Or)_2_(bipy)]·3H_2_O, (**2**), whose properties and phase transition are the main subject of this report. Compound (**1**) is chemically isostructural with its nickel analogue (Falvello *et al.*, 2007 ▸), which was prepared using the metal chloride (NiCl_2_) as starting material instead of the carbonate. The use of the metal carbonate instead of the chloride reduces the amount of ^*n*^Bu_4_NOH needed and eliminates the formation of residual products (^*n*^Bu_4_NCl).

### Crystal structure of compound (1)   

**Figure 1:**
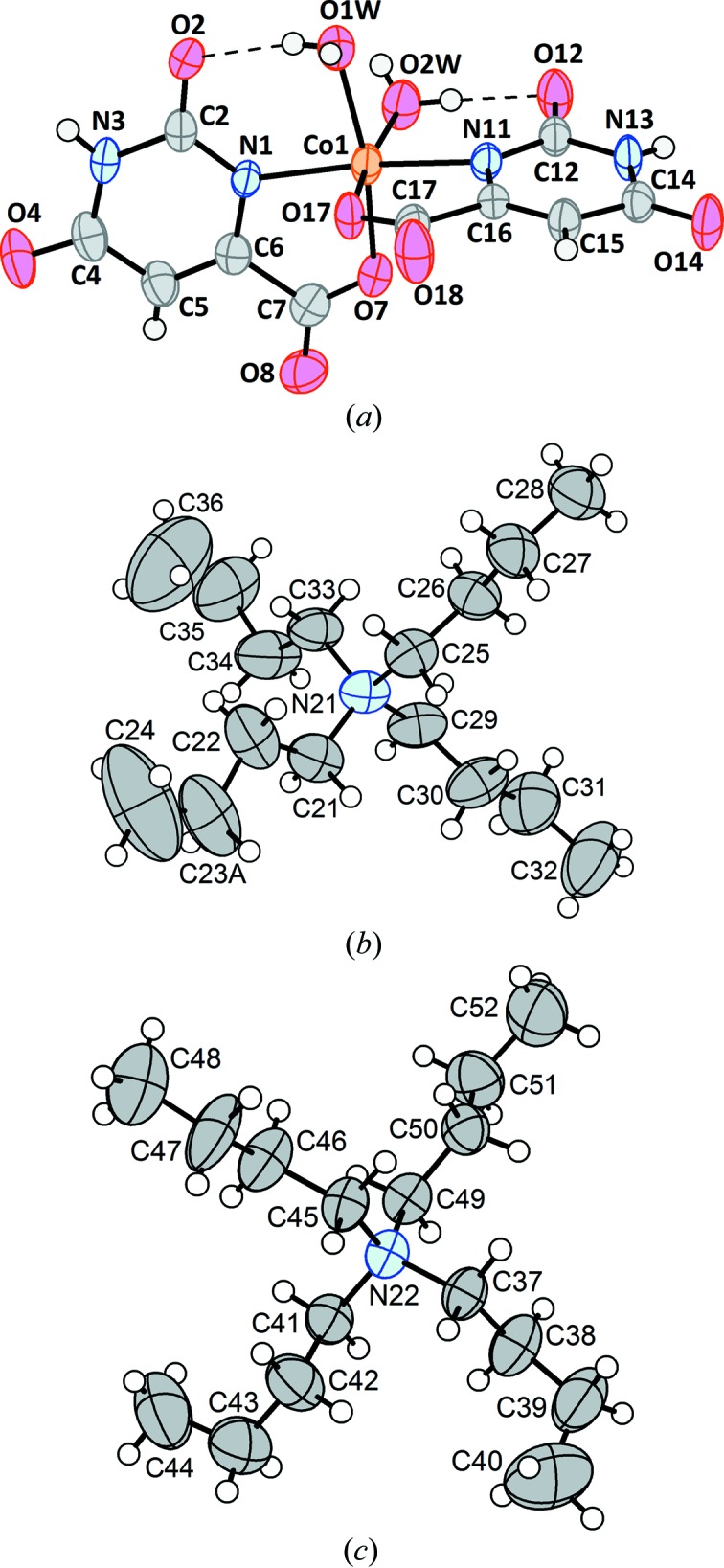
(*a*) The anion, (*b*) the cation centred at N21 and (*c*) the cation centred at N22 from the crystal structure of (^*n*^Bu_4_N)_2_[*cis*-Co(Or)_2_(H_2_O)_2_]·1.8H_2_O, (**1**). In all three drawings, non-H atoms are represented by their 50% probability displacement ellipsoids. In part (*b*), the minor-disordered congener at the C23 site has been omitted, along with the corresponding H atoms.

Crystals of compound (**1**) are isomorphous with the analogous Ni complex, whose structure has been discussed in detail (Falvello *et al.*, 2007 ▸). The distorted octa­hedral environment of atom Co1 (Fig. 1 ▸) has the two aqua ligands *cis* to each other, and the two chelating Or^2−^ ligands are disposed such that their coordinated N atoms are mutually *trans* and their ligated carboxyl­ate O atoms *cis*. As was discussed for the corresponding Ni complex, crystallization from an environment poor in hydrogen-bonding possibilities leads to isolation of the *cis* isomer, in which two intra­molecular hydrogen bonds add stability to the structure. In the absence of other hydrogen-bonding partners, (**1**) also enters into self-complementary aggregation patterns, namely an 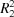
(8) inter­action with the N13—H13 group as donor and atom O14 at (−*x* + 1, −*y* + 1, −*z* + 1) as acceptor, and an 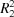
(12) cycle involving the N3—H3 group and atom O17 – that is, two different orotate ligands – and the mol­ecule at (−*x*, −*y* + 1, −*z*) (see Fig. S3 in the supporting information). The hydrogen-bonded chain thus formed propagates along [101].

### Crystal structures of (2)   

**Figure 2:**
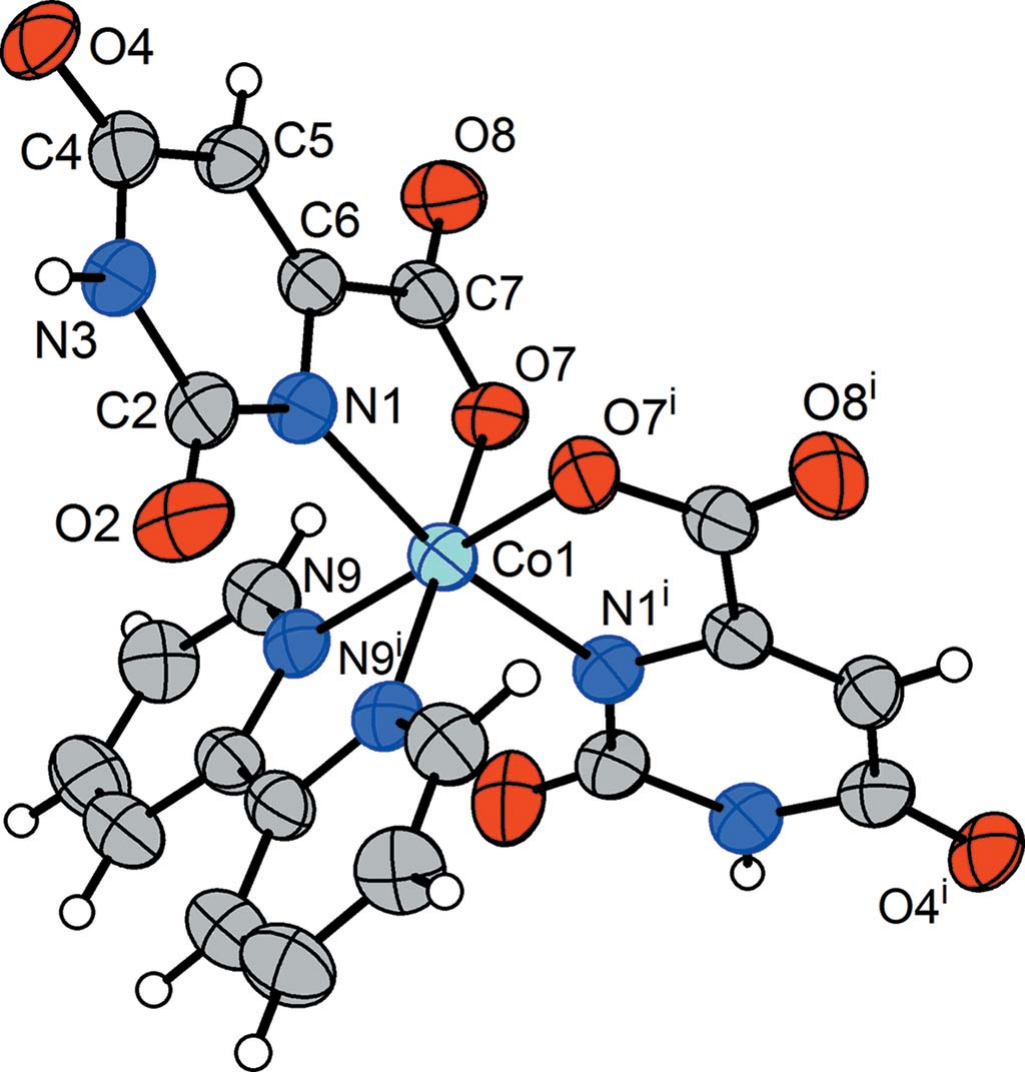
The anion from the monoclinic crystal structure of (NBu_4_)[*cis*-Co(Or)_2_(bipy)]·3H_2_O, (**2a**). Non-H atoms are represented by their 50% probability displacement ellipsoids. [Symmetry code: (i) −*x* + 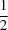, *y*, −*z* + 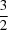.]

In the monoclinic room-temperature form of compound (**2**) – we refer to this analysis of the as-prepared crystal as structure (**2a**) – the ^*n*^Bu_4_N^+^ cation and the six-coordinate Co^III^ complex both reside on crystallographic twofold axes, as does one of the two independent unligated water mol­ecules. The anionic six-coordinate complex (Fig. 2 ▸) presents an arrangement of orotate ligands similar to that found for Co^II^ complex (**1**), with the coordinated N1 atoms of the two ligands *trans* to each other and the coordinated carboxyl­ate O7 atoms mutually *cis*. The chelating bipy ligand occupies the remaining two coordination sites. Except for the differences in the Co1—N and Co1—O bond lengths that accompany the change of oxidation state of the Co centre, the geometries of complex salts (**1**) and (**2**) are similar.

**Figure 3:**
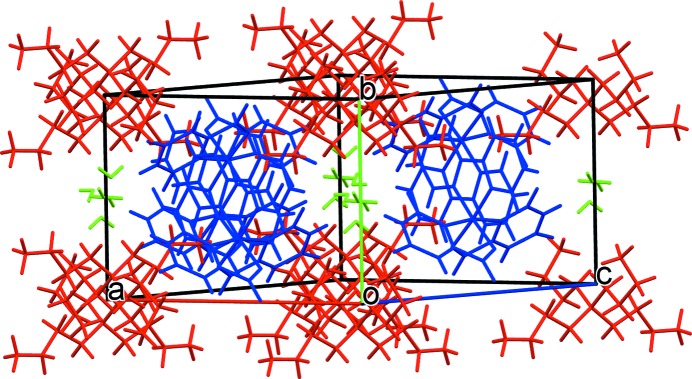
The packing in monoclinic (^*n*^Bu_4_N)[*cis*-Co(Or)_2_(bipy)]·3H_2_O, (**2a**), showing the separation of hydro­philic and hydro­phobic fragments into layers perpendicular to [010]. Blue represents [*cis*-Co(Or)_2_(bipy)]^−^, red ^*n*^Bu_4_N^+^ and green H_2_O.

A narrow channel parallel to [101] and at a height of *y* = 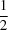 is occupied by ordered water mol­ecules that act as hydrogen-bond donors and acceptors in inter­actions with the orotate ligands. There is one relatively weak hydrogen bond between the two free water mol­ecules, but hydrogen bonding involving only water mol­ecules along the water-occupied channel is not an important feature of this structure. This can be contrasted to the water wire that has been found to be a proton conductor in a mol­ecular crystal involving a Mn^II^ citrate cubane polymer (Capelli *et al.*, 2013 ▸). In (**2a**), units of the Co^III^ complex occupy a slab perpendicular to the *b* axis (Fig. 3 ▸), and hydrogen bonding, albeit weak, joins these anions (blue in the figure) and the two independent water mol­ecules (green) into a sheet. This sheet and the hydro­phobic cations (red) are segregated into alternating layers along the *b* axis, with the cations in a layer centred at *y* = 0.0.

**Figure 4:**
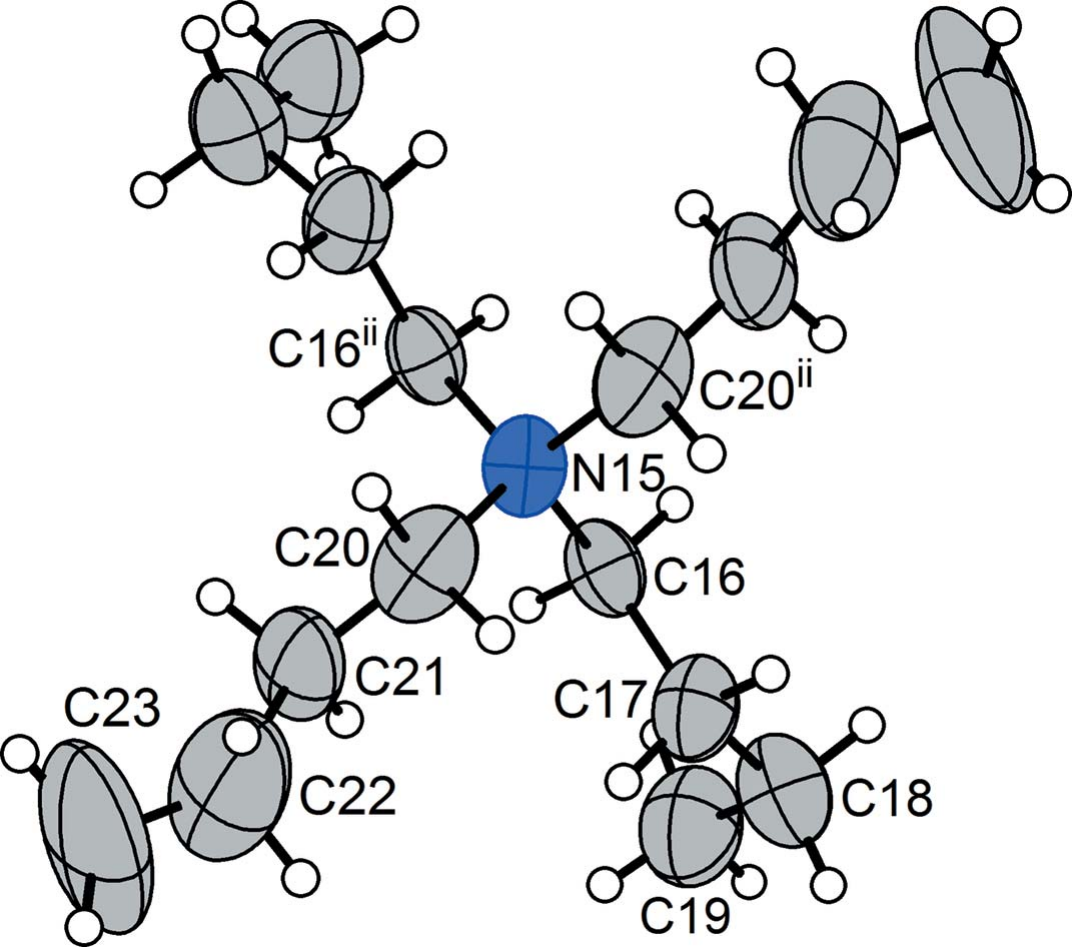
The ^*n*^Bu_4_N^+^ cation in monoclinic (**2a**), with non-H atoms represented by 50% probability displacement ellipsoids. The prolate ellipsoids for atoms C22 and C23 can be seen. [Symmetry code: (ii) −*x* + 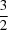, *y*, −*z* + 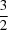.]

The ^*n*^Bu_4_N^+^ cation in (**2a**), which is the protagonist of the phase transition that befalls this crystal, merits a mention. At room temperature, two of the terminal ethyl fragments of the ^*n*^Bu groups have their displacement ellipsoids elongated in a fashion that suggests concerted motion of this group, most likely libration in what is a typical example of dynamic disorder. This can be seen in Fig. 4 ▸, where the displacement ellipsoids for atoms C22 and C23, and their symmetry relatives, are notably more prolate, with transverse elongation, than those of the other C atoms of the ^*n*^Bu chains. (When a single atomic position is modelled for sites such as these, they are not flagged as disordered entities in the CSD.)

**Figure 5:**
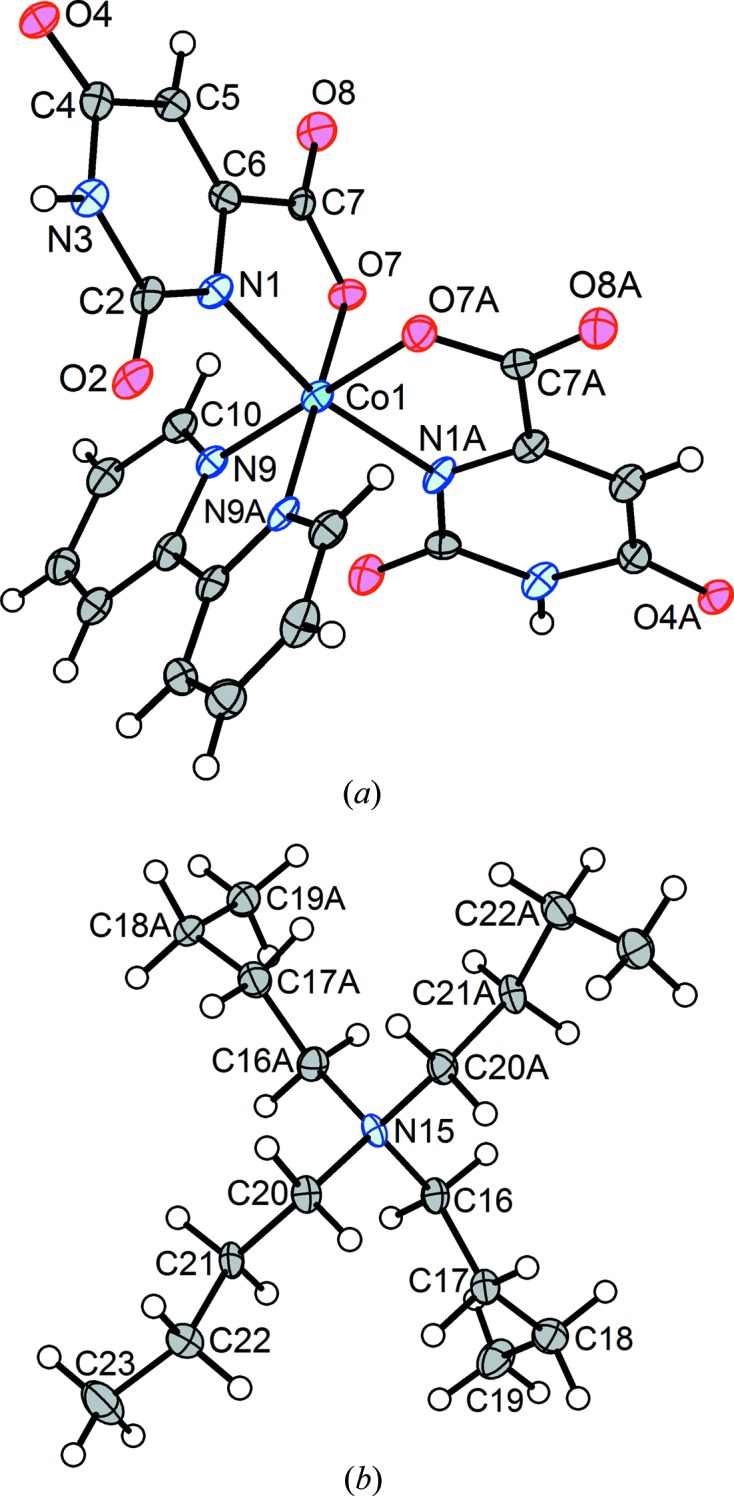
(*a*) The [*cis*-Co(Or)_2_(bipy)]^−^ anion and (*b*) the ^*n*^Bu_4_N^+^ cation in (**2b**), with non-H atoms represented by 50% probability displacement ellipsoids in both parts.

**Figure 6:**
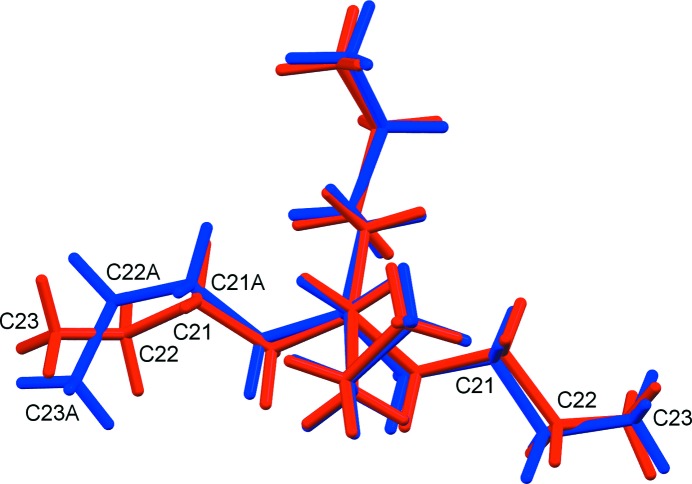
Superposition of the ^*n*^Bu_4_N^+^ cations from monoclinic (**2a**) (red) and triclinic (**2b**) (blue). The two sets of atoms labelled C22 and C23 are related by symmetry in (**2a**), while C22*A* and C23*A* are at the new positions for one of these fragments in the triclinic structure.

When a crystal of compound (**2**) is cooled to 100 K, it undergoes a reversible transition to a triclinic structure, *i.e.* (**2b**), that is a minor modification of the monoclinic room-temperature structure, with the only significant difference at the mol­ecular level being a separation of the prolate symmetry relatives of atoms C22 and C23 into fragments not related by the twofold axis. As is generally expected for a conservative monoclinic-to-triclinic transformation, the crystal becomes a twin. The structure was solved *ab initio* and refined using the usual protocol for so-called ‘nonmerohedral twins’ (Herbst-Irmer, 2016 ▸), with the diffraction data integrated using two orientation matrices for the two twin components, and with overlapped reflections separated as well as the software is able to do. The asymmetric unit in (**2b**) comprises one full cation, one full anion and three water mol­ecules. The reference asymmetric unit for (**2b**) was chosen to correspond as closely as possible to that of monoclinic (**2a**), with ‘A’ appended to the names of the newly independent atoms – those that are related to the reference asymmetric unit by a twofold axis in the monoclinic structure. The complex anion in (**2b**) is essentially identical to that in (**2a**) (Fig. 5 ▸). It can be seen that the displacement ellipsoids for both ions behave well in (**2b**), except for effects attributable to the twinning. The ^*n*^Bu_4_N^+^ cation is conformationally different at one extreme of one of the ^*n*^Bu chains. Specifically, the newly independent terminus of the chain at C22*A*/C23*A* has been reoriented to give a *syn* conformation about the C21*A*—C22*A* bond, while the original chain at C22/C23 is still *anti* in the triclinic structure, as it was in the monoclinic mother phase. Fig. 6 ▸ shows superposed drawings of the cations from (**2a**) (red) and (**2b**) (blue). Three of the ^*n*^Bu groups are almost identical in the two structures. The groups that had large prolate displacement ellipsoids (C22 and C23 at the right of the figure) are those that have segregated conformationally as indicated above.

The general features of the packing in (**2b**) (Fig. S4 in the supporting information) are essentially unchanged from the original structure (**2a**). The major features of the extended structure are a segregation of the hydro­phobic cation and more hydro­philic anion layers, along with a line of water mol­ecules weakly hydrogen bonded to the anions, running along [101].

The quality indicators for the refinement of (**2b**) are not ideal (Table 2 ▸). We use this analysis to demonstrate that the transformation has taken place under the conditions described and to establish its nature. A better refinement was achieved for triclinic (**2e**) (see below). In addition, more accurate geometrical parameters for the anion and cation are available from the refinements of monoclinic (**2a**), (**2c**) and (**2d**). Regarding the ^*n*^Bu_4_N^+^ cation, its geometries have already been established in some 4742 previously published structure analyses recorded in the CSD.

That the original structure (**2**) is monoclinic with dynamic disorder and not triclinic without disorder and with only a slight deviation from monoclinic symmetry is clear from the fact that a transition to triclinic, accompanied by twinning, occurs on lowering the temperature. That transition, directly observable in the diffraction itself, is reversed when the temperature is raised again, and a single-domain monoclinic structure can be analyzed from the same sample after cycling the temperature. Clearer evidence for dynamic disorder is presented below.

### Characterization of the phase transition by thermal analysis   

**Figure 7:**
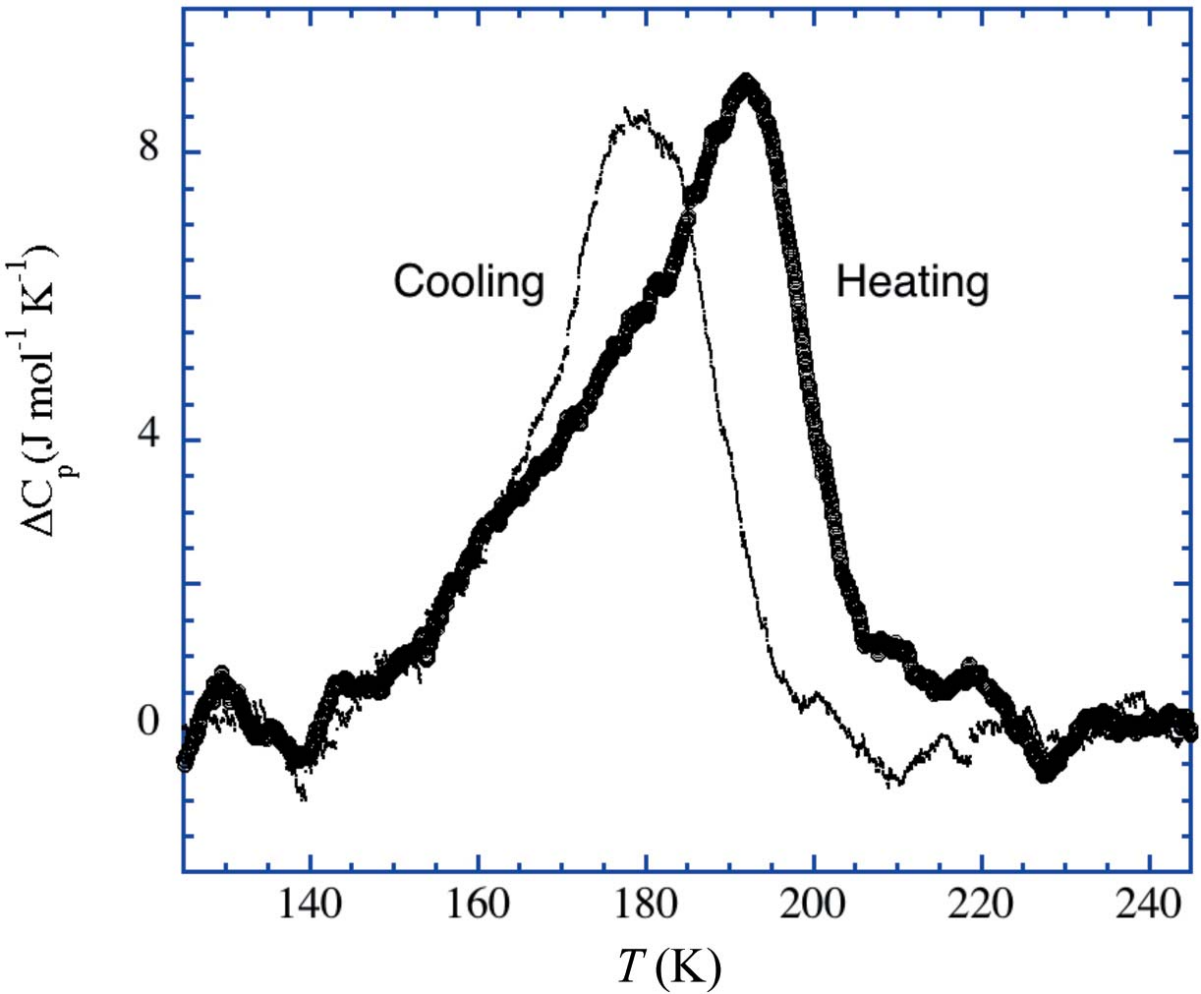
Heat-capacity anomalies measured by DSC for both heating (thick line) and cooling (thin line) thermograms.

The heat-capacity anomalies determined by differential scanning calorimetry (DSC) are shown in Fig. 7 ▸ for both heating and cooling thermograms. These small broad anomalies present their maximum temperatures at around 192 and 177 K, respectively, highlighting the first-order character of the transition, with a hysteresis of 13 K at a 10 K min^−1^ scan rate. These temperatures and the hysteresis are in agreement with the results of the X-ray diffraction measurements, which also indicated that the transition occurred roughly within the temperature range 170–200 K.

The calculated enthalpy (entropy) contents, after sub­tracting the baseline, are roughly 0.28 (1.56 J mol^−1^ K^−1^) and 0.21 kJ mol^−1^ (1.23 J mol^−1^ K^−1^) for the heating and cooling anomalies, respectively. These values are small, also in agreement with the diffraction results, which reveal that the structural changes consist of minor conformational adjustments at the periphery of the ^*n*^Bu_4_N^+^ cation.

### Arrested phase transition   

As just described, DSC established more accurately the temperature range in which the transition of (**2**) from monoclinic to triclinic takes place. The heat-capacity anomalies, with maxima at 177 (cooling) and 192 K (heating), point to a first-order transition with hysteresis. On exploring this reversible phase transition further, using single-crystal diffraction with a fresh crystal, a more complex behaviour was revealed.

First, a unit-cell determination at *T* = 220 K confirmed that the crystal was monoclinic with the known cell of (**2a**). Then axial photos of [010] were used to follow the transformation accompanied by twinning as the temperature was lowered to 170 K in increments of 10 or 20 K (see *Experimental* for details). The temperature was then raised in similar increments and axial photos revealed that the spot-splitting that had accompanied the transformation to triclinic (**2b**) was reversed as the temperature was raised and the original monoclinic structure was restored.

**Figure 8:**
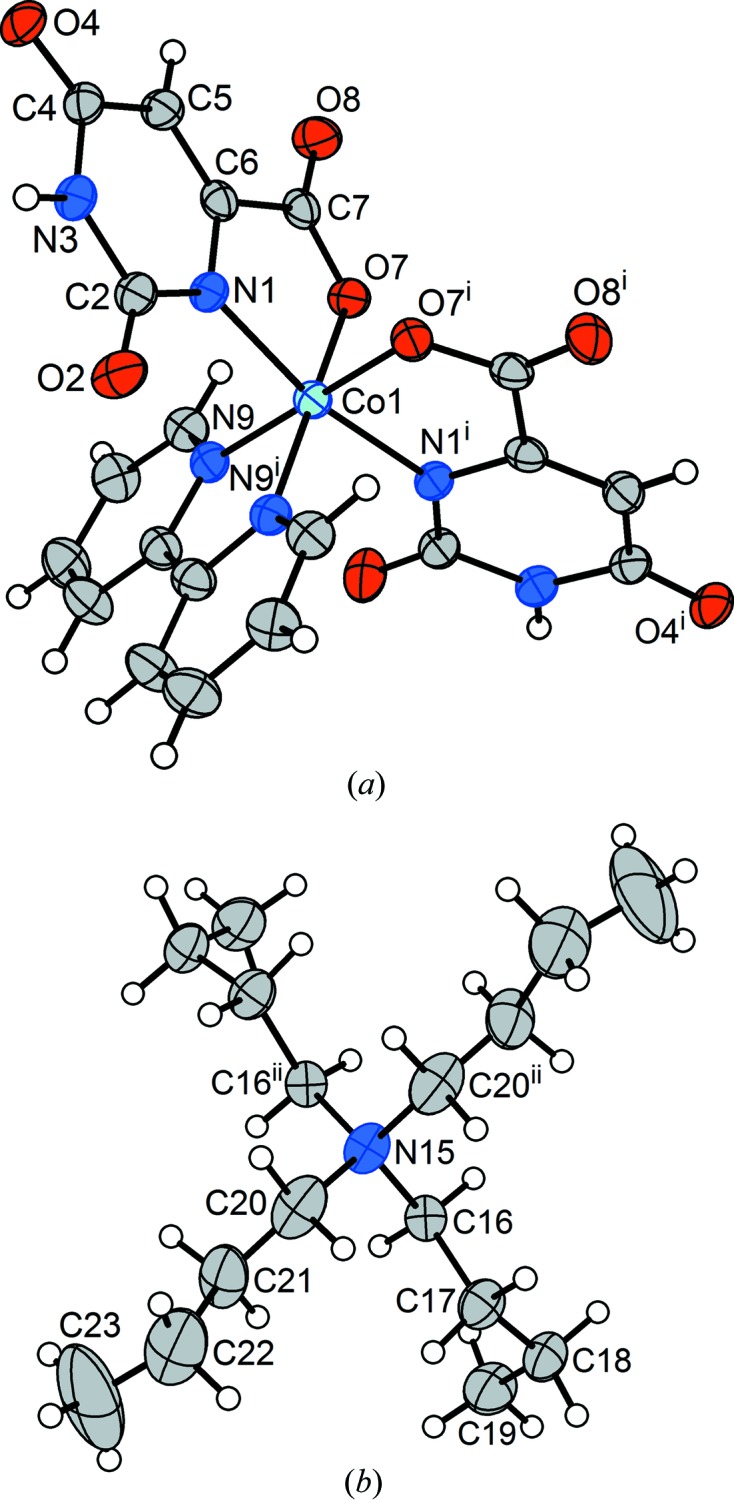
(*a*) The anion and (*b*) the ^*n*^Bu_4_N^+^ cation from monoclinic form (**2c**) of (^*n*^Bu_4_N)[*cis*-Co(Or)_2_(bipy)]·3H_2_O following one full cycle through the phase transition. Non-H atoms are represented by 50% probability displacement ellipsoids in both parts. [Symmetry codes: (i) −*x* + 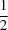, *y*, −*z* + 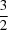; (ii) −*x* + 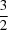, *y*, −*z* + 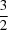.]

A complete structure determination, (**2c**), was carried out after the crystal had been warmed again to *T* = 220 K, and the monoclinic structure was confirmed (Fig. 8 ▸) to be isostructural with (**2a**). Following this full cycle of the transition, there were minor indications that the crystal was not of quite the high quality that it had originally possessed – there were a number of inconsistent symmetry equivalents, and the unit-cell angles α and γ, when not constrained to their monoclinic values of 90°, refined to values of 90.168 (2) and 90.117 (2)°, respectively. Nevertheless, the structure was developed and refined to the residuals given for (**2c**) in Table 2 ▸. As an indicator of the quality of the data, we note that the H atoms of the unbound water mol­ecules were located in a difference map and refined freely, including their isotropic displacement parameters. Except for effects that can be attributed to the difference in temperature, the structure of (**2c**) is identical to that of the near-room-temperature structure (**2a**).

**Figure 9:**
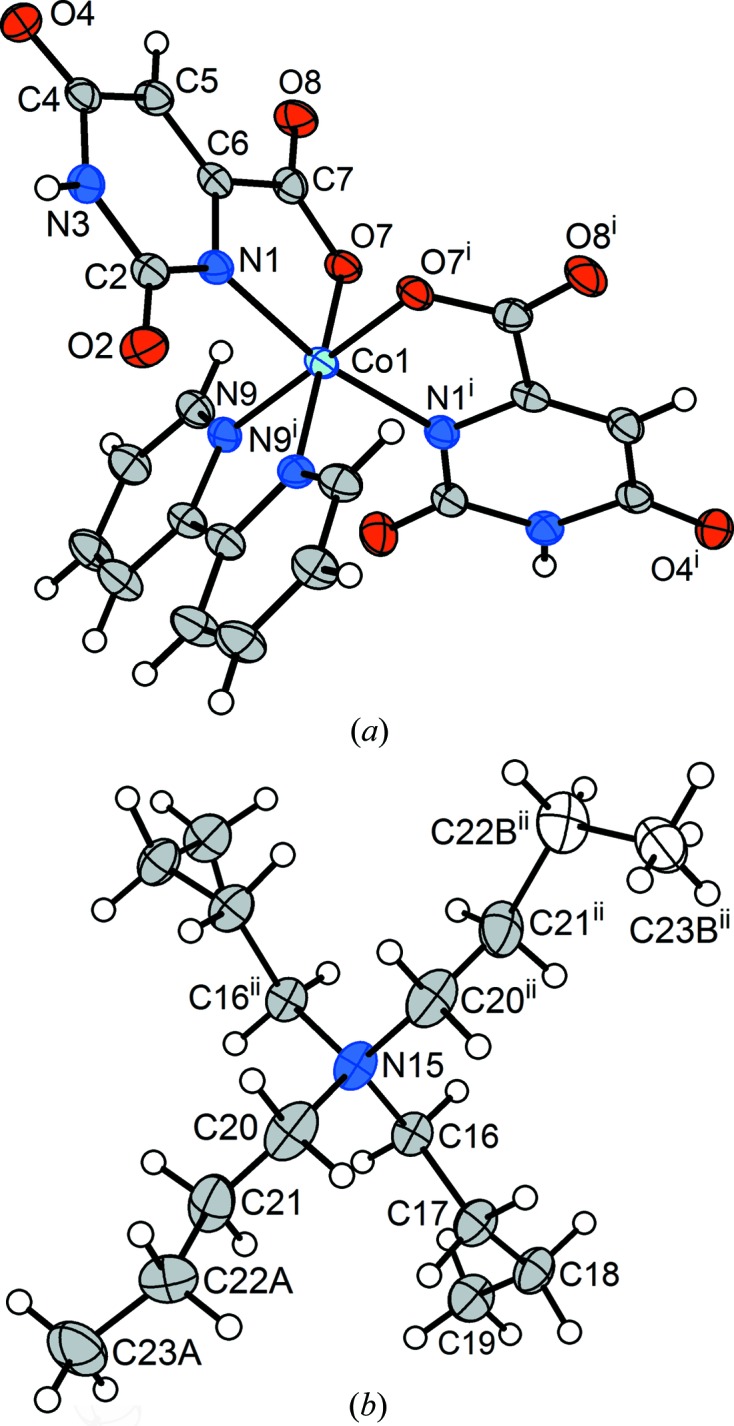
(*a*) The anion and (*b*) the ^*n*^Bu_4_N^+^ cation from monoclinic phase (**2d**) of the multicrystal of (^*n*^Bu_4_N)[*cis*-Co(Or)_2_(bipy)]·3H_2_O. Non-H atoms are represented by 50% probability displacement ellipsoids in both parts. For the ^*n*^Bu_4_N^+^cation in part (*b*), the major-disorder component (C22*A*—C23*A*) is shown for one ^*n*^Bu group and the minor component (C22*B* ^ii^—C23*B* ^ii^) is shown for its symmetry relative. [Symmetry codes: (i) −*x* + 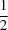, *y*, −*z* + 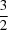; (ii) −*x* + 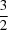, *y*, −*z* + 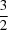.]

**Figure 10:**
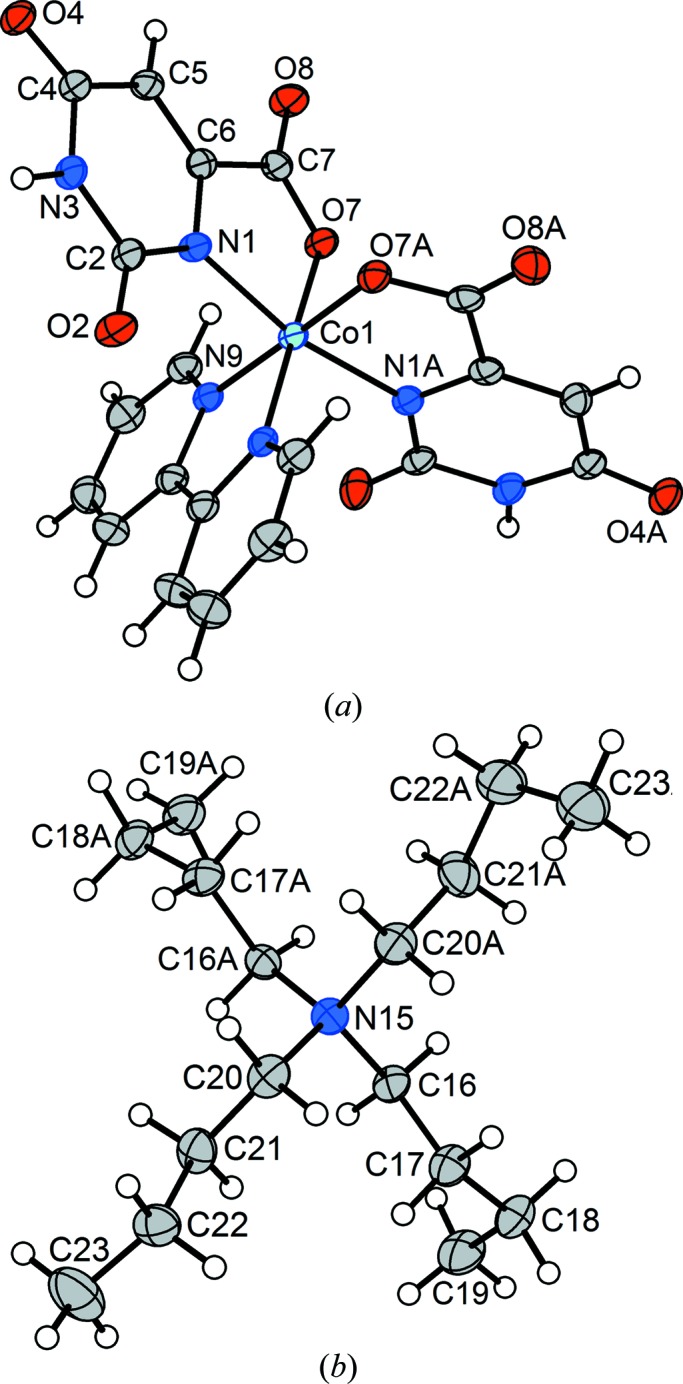
(*a*) The [*cis*-Co(Or)_2_(bipy)]^−^ anion and (*b*) the ^*n*^Bu_4_N^+^ cation from triclinic phase (**2e**) of the multicrystal of (^*n*^Bu_4_N)[*cis*-Co(Or)_2_(bipy)]·3H_2_O. Non-H atoms are represented by 50% probability displacement ellipsoids in both parts.

After this one full cycle of the transition from monoclinic to triclinic and back, lowering the temperature again, directly, to *T* = 170 K, produced an arrested form of the transition, in which about one-half of the sample once again changed to the triclinic form and the rest remained in the monoclinic structure. (The temperature was lowered from 220 to 170 K over a period of several minutes and then held at *T* = 170 K for 4 h before the diffraction measurements commenced.) To our knowledge, a result of this entirely unexpected nature has not previously been characterized in detail for a mol­ecular crystal. A case of several structures being characterized from the same sample has been reported recently (Aromí *et al.*, 2016 ▸). The difference in the present case is that the crystal remained stable with its two components at 170 K and, furthermore, despite a good deal of reflection overlap it was possible to isolate complete redundant individual data sets for both components using in-house data. Both the monoclinic (**2d**) (Fig. 9 ▸) and triclinic (**2e**) (Fig. 10 ▸) phases gave high-quality refinements in which the positional parameters of the H atoms attached to free water were refined freely. (The isotropic displacement parameters of these H atoms were constrained to 1.2 times *U*_eq_ of their bonding partners.)

The diffraction pattern for this final set of measurements revealed just two principal phases, one monoclinic and one triclinic. It appears that the twinning of the triclinic phase that would be expected for a clean transition was not a major feature in this case.

The nature of the phase change can be understood readily with reference to Table 3 ▸, which collects the relevant geometrical parameters for the ^*n*^Bu group that changes, namely C20—C21—C22—C23. In the monoclinic structure, it is related by a twofold axis to another such chain and it is highly likely that both congeners are affected by dynamic disorder (*vide infra*). In the triclinic structure, the second congener, C20*A*—C21*A*—C22*A*—C23*A*, is not related by symmetry to the first and it is the second congener that undergoes the change. The torsion angle C20*A*—C21*A*—C22*A*—C23*A*, which in the monoclinic structure describes an *anti* conformation (Table 3 ▸), is modified to a *syn* arrangement in the triclinic structure. The base unit, *i.e.* C20—C21—C22—C23, retains its *anti* descriptor in the triclinic structure, where no disorder is evident.

Monoclinic phase (**2d**) of the multicrystal that results from the arrested transition gives a structure analysis at *T* = 170 K with a major component that is nearly identical – but not rigorously so – to those obtained for the monoclinic phases at *T* = 277 (**2a**) and 220 K (**2c**). A telling difference involves the unique ^*n*^Bu group that suffers disorder at higher temperature (C20–C23, Fig. 4 ▸). Disorder is reflected in the principal mean-square displacement amplitudes (MSDA) for atoms C22 and C23 (Table 3 ▸). As is also clear from Table 3 ▸, the foreshortening of the ‘apparent’ C—C distance that accompanies librational disorder is pronounced at *T* = 277 K for (**2a**), significant but less exaggerated at 220 K for (**2c**) and observable but small at 177 K for (**2d**). Such variation with temperature discriminates between dynamic and static disorder, and is a strong indicator in this case for dynamic disorder. Being able to make this determination is one of several reasons for not restraining the terminal C—C bond length in the higher-temperature determination.

Possibly more intriguing is that the monoclinic structure (**2d**) at *T* = 170 K, derived from the sample after the arrested phase transition, has a minor-disordered component with one ^*n*^Bu group in a *syn* conformation, as in the triclinic structure that results from the phase transition. We refrain from drawing speculative conclusions, but it may be that the first step in the phase transition is a conformational change in the affected ^*n*^Bu group, and that this is then followed by the global change of the sample to the triclinic phase.

The arrested transition, which may well be fortuitous, also permits a more exact comparison between the two phases than is often the case with transitions, because it was possible to characterize the two phases at the same temperature. In actual fact, all five structural results can be superimposed quite well – anion, cation and inter­stitial water – except for terminal atoms C22*A* and C23*A*, which upon ordering mark the difference in the triclinic phase.

## Concluding comments   

We refer to the partially executed change from monoclinic to triclinic in this case as an *arrested phase transition*. Given that the transition proceeds to completion in both directions in the first cycle, we conclude that in the second cycle, defects are responsible for blocking the advance of the transformation following normal nucleation. We are not attempting to coin a term for this phenomenon. We note that the term ‘arrested phase transition’ was used in the *Abstract* of an article by Xu & Veblen (1995 ▸) describing a transition in haüyne that does not go to completion. The term ‘arrested solid–solid phase transition’ was also used in the *Title*, but not the text, of an article describing displacements of phase-transition temperatures or pressures in CdS nanocrystals, as compared to the bulk material, as a result of surface characteristics (Haase & Alivisatos, 1992 ▸).

The phase transition from a dynamically disordered monoclinic room-temperature structure to an ordered but twinned triclinic structure at low temperature underlines some counterintuitive features of this type of system. The room-temperature structure, and the structure to temperatures as low as 220 K, have excellent quality indicators and betray the dynamic disorder only in the displacement parameters of the affected atoms and in the apparently foreshortened bond distance at the end of one of the unique ^*n*^Bu groups.

For compound (**2**), unlike what is found for most mol­ecular crystalline systems, but not unprecedented or completely unexpected, lowering the temperature gives a decidedly worse diffraction pattern because of the twinning that accompanies the conservative symmetry-lowering transition. It is known that this occurs for some crystals, and this is a phenomenon that is worth keeping in mind when an otherwise apparently good crystal that is abruptly subjected to low temperatures displays a surprisingly poor diffraction pattern.

## Supplementary Material

Crystal structure: contains datablock(s) 1, 2a, 2b, 2c, 2d, 2e, global. DOI: 10.1107/S2053229617010841/sk3662sup1.cif

Structure factors: contains datablock(s) 1. DOI: 10.1107/S2053229617010841/sk36621sup2.hkl

Structure factors: contains datablock(s) 2a. DOI: 10.1107/S2053229617010841/sk36622asup3.hkl

Structure factors: contains datablock(s) 2b. DOI: 10.1107/S2053229617010841/sk36622bsup4.hkl

Structure factors: contains datablock(s) 2c. DOI: 10.1107/S2053229617010841/sk36622csup5.hkl

Structure factors: contains datablock(s) 2d. DOI: 10.1107/S2053229617010841/sk36622dsup6.hkl

Structure factors: contains datablock(s) 2e. DOI: 10.1107/S2053229617010841/sk36622esup7.hkl

Supporting information file. DOI: 10.1107/S2053229617010841/sk36621sup8.mol

Supporting information file. DOI: 10.1107/S2053229617010841/sk36622asup9.mol

IR spectra and packing plots. DOI: 10.1107/S2053229617010841/sk3662sup10.pdf

CCDC references: 1560743, 1560742, 1560741, 1560740, 1560739, 1560738

## Figures and Tables

**Table 1:** Crystal data and refinement quality indicators for the structure analysis of (^*n*^Bu_4_N)_2_[*cis*-Co(Or)_2_(H_2_O)_2_]·1.8H_2_O, (**1**)

Structure	(**1**)
CCDC reference	1560738
Formula	C_42_H_80_CoN_6_O_10_·1.8H_2_O
Formula weight	920.47
Crystal	1
Crystal history	as prepared
*T* (K)	295 (2)
Crystal condition	single
Crystal system	triclinic
Space group	*P* 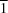
*Z*	2
H^*a*^ (H_2_O, N—H) located and refined	mixed: some water H located and refined (*xyz* and *U*_iso_), some not located
Resolution^*b*^ (Å)	0.77
No. data, total	26453
Independent data	11477
*R*_int_	0.0343
Parameters	597
Restraints	8
*R*1 [*F* ^2^ > 2σ(*F* ^2^)]	0.0469
*wR*2 (all data used)	0.1034
Quality-of-fit	1.033
*a* (Å)	12.3630 (4)
*b* (Å)	12.6281 (5)
*c* (Å)	16.3765 (6)
α (°)	89.948 (3)
β (°)	95.460 (3)
γ (°)	96.455 (3)
*V* (Å^3^)	2528.86 (16)
Δρ_max_,Δρ_min_ (e Å^−3^)	0.477, −0.319

Notes: (*a*) H atoms bonded to O or N atoms. Whether or not these H atoms are located and refined is an indicator for the reliability of the structure analysis. (*b*) Resolution is estimated as the minimum Bragg spacing to which data are at least 95% complete, based on the Laue group. Computer programs: *CrysAlis CCD* (Oxford Diffraction, 2006 ▸), *CrysAlis RED* (Oxford Diffraction, 2006 ▸), *SIR92* (Altomare *et al.*, 1994 ▸), *SHELXL2014* (Sheldrick, 2015 ▸) and *DIAMOND* (Brandenburg & Putz, 2004 ▸).

**Table 2:** Crystal data and refinement quality indicators for the five determinations of the structure of (^*n*^Bu_4_N)[Co(Or)_2_(bipy)]·3H_2_O, (**2**) Mol­ecular formula C_36_H_54_CoN_7_O_11_ or (C_16_H_36_N)[Co(C_5_H_2_N_2_O_4_)_2_(C_10_H_8_N_2_)_2_]·3H_2_O; *M*_r_ = 819.79.

Structure	(**2a**)	(**2b**)	(**2c**)	(**2d**)	(**2e**)
CCDC reference	1560739	1560740	1560741	1560742	1560743
Crystal	1	1	2	2	
Crystal history	as prepared	after one transition monoclinic to triclinic	following one full cycle monoclinic to triclinic to monoclinic	following 1.5 full cycles, monoclinic to triclinic to monoclinic to mixed monoclinic/triclinic	
*T* (K)	277 (1)	100 (1)	220 (1)	170 (1)	170 (1)
Crystal condition	single	twin	single	multicrystal	
Crystal system	monoclinic	triclinic	monoclinic	monoclinic	triclinic
Space group	*P*2/*n*	*P* 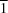	*P*2/*n*	*P*2/*n*	*P* 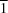
*Z*	2	2	2	2	2
H^*a*^ (H_2_O, N—H) located and refined	*xyz* and *U*_iso_ refined	no	*xyz* and *U*_iso_ refined	*xyz* refined and *U*_iso_ constrained	*xyz* refined and *U*_iso_ constrained
Resolution^*b*^ (Å)	0.84	0.78	0.77	0.77	0.84
No. data, total	10924	15136	21720	22527	25546
Independent data	4564	15136	4673	4643	6796
*R*_int_	0.0664	twin^*c*^	0.0499	0.1647	0.1521
Parameters	264	501	264	282	524
Restraints	0	0	0	39	0
*R*1 [*F* ^2^ > 2σ(*F* ^2^]	0.0549	0.0949	0.0480	0.0664	0.0617
*wR*2 (all data used)	0.1100	0.2481	0.1294	0.1646	0.1608
Quality-of-fit	1.021	1.439	1.070	1.075	1.052
*a* (Å)	13.1679 (12)	12.9054 (8)	13.0259 (4)	13.0080 (8)	13.0155 (15)
*b* (Å)	9.3413 (9)	9.3791 (8)	9.3504 (3)	9.3320 (6)	9.4028 (14)
*c* (Å)	16.3388 (14)	16.1290 (12)	16.3308 (5)	16.3753 (12)	16.2640 (17)
α (°)	90	88.724 (6)	90	90	88.794 (11)
β (°)	102.669 (9)	102.898 (6)	103.847 (3)	104.364 (7)	103.054 (9)
γ (°)	90	88.528 (6)	90	90	88.687 (11)
*V*, Å^3^	1960.8 (3)	1901.6 (2)	1931.24 (11)	1925.7 (2)	1937.8 (4)
Δρ_max_,Δρ_min_ (e Å^−3^)	0.478, −0.309	2.876, −0.771	1.044, −0.915	0.741, −0.731	1.630, −0.899

Notes: (*a*) H atoms bonded to O or N atoms. Whether or not these H atoms are located and refined is an indicator for the reliability of the structure analysis. (*b*) Resolution is estimated as the minimum Bragg spacing to which data are at least 95% complete, based on the Laue group. (*c*) Structure (**2b**) was refined using data from two domains in the same refinement. Traditional data merging was not performed. Computer programs: *CrysAlis CCD* (Oxford Diffraction, 2006 ▸), *CrysAlis RED* (Oxford Diffraction, 2006 ▸, 2009 ▸), *CrysAlis PRO* (Agilent, 2011 ▸), *SIR92* (Altomare *et al.*, 1994 ▸), *SHELXL2014* (Sheldrick, 2015 ▸) and *DIAMOND* (Brandenburg & Putz, 2004 ▸).

**Table 3:** Comparison of geometric parameters (Å, °) for the variable ^*n*^Bu group in the structures of (^*n*^Bu_4_N)[Co(Or)_2_(bipy)]·3H_2_O, (**2**) Codes: m = monoclinic and t = triclinic.

Structure	(**2a**) (m)	(**2b**) (t)	(**2c**) (m)	(**2d**) (m)^*b*^	(**2e**) (t)
*T* (K)	277 (1)	100 (1)	220 (1)	170 (1)	170 (1)
C21—C22	1.552 (5)	1.52 (3)	1.532 (4)	1.544 (5)	1.535 (5)
C21*A*—C22*A*		1.54 (3)			1.546 (5)
C22—C23^*a*^	1.182 (6)	1.52 (3)	1.321 (7)	1.512 (7)	1.501 (6)
C22*A*—C23*A*		1.49 (3)			1.498 (6)
C22 principal MSDA (Å^2^)	0.3296, 0.1017, 0.0544	0.0281, 0.0246, 0.0180	0.1866, 0.0627, 0.0301	0.0533, 0.0358, 0.0189	0.0440, 0.0321, 0.0267
C23 principal MSDA (Å^2^)	0.5481, 0.1254, 0.0527	0.0562, 0.0307, 0.0182	0.4189, 0.0769, 0.0353	0.0778, 0.0558, 0.0267	0.0881, 0.0524, 0.0279
C22*A* principal MSDA (Å^2^)		0.0343, 0.0250, 0.0153			0.0613, 0.0428, 0.0306
C23*A* principal MSDA (Å^2^)		0.0339, 0.0264, 0.0179			0.0682, 0.0462, 0.0351
N15—C20—C21—C22	168.3 (3)	166.1 (17)	168.7 (2)	170.1 (3)	166.7 (3)
N15—C20*A*—C21*A*—C22*A*		164.2 (17)			163.4 (3)
C20—C21—C22—C23	150.6 (8)	174.9 (19)	164.5 (6)	173.8 (4)	173.7 (3)
C20*A*—C21*A*—C22*A*—C23*A*		68 (3)			68.6 (5)

Notes: (*a*) where severely affected by dynamic disorder, as in (**2a**) and (**2c**), these values are referred to as ‘apparent distances’. (*b*) The parameters given for monoclinic (**2d**) are those of the major-disorder component, *i.e.* C20—C21—C22*A*—C23*A*.
