# Supplementary material for: A phase transition caught in mid-course: independent and concomitant analyses of the monoclinic and triclinic structures of (nBu4N)[Co(orotate)2(bipy)]·3H2O
Source: Acta Crystallogr C Struct Chem. 2017 Aug 17;73(Pt 9):731–42. doi: 10.1107/S2053229617010841 (PMC5601254; doi:10.1107/S2053229617010841)
Supplement: Supplementary file 10 [file c-73-00731-sup10.pdf]

STRUCTURAL CHEMISTRY

**Volume 73 (2017)**

**Supporting information for article:**

# A phase transition caught in mid-course: independent and concomitant analyses of the monoclinic and triclinic structures of ($^{n}$Bu4N)[Co(orotate)2(bipy)]·3H2O

**Miguel Castro, Larry R. Falvello, Elena Forcén-Vázquez, Pablo Guerra, Nuha A. Al-Kenany, Gema Martínez and Milagros Tomás**

# A phase transition caught in mid-course: Independent and concomitant analyses of the monoclinic and triclinic structures of ($^{n}$Bu$_4$N)[Co(orotate)$_2$(bipy)]·3$H_2O$

Authors

**Miguel Castro[a], Larry R. Falvello[b]*, Elena Forcén-Vázquez[b], Pablo Guerra[b], Nuha Mushale Aref[b], Gema Martínez[b] and Milagros Tomás[c]***

[a]Departamento de Ciencia y Tecnología de Materiales y Fluidos, Escuela de Ingeniería y Arquitectura – Instituto de Ciencia de Materiales de Aragón (ICMA), University of Zaragoza - CSIC, María de Luna 3, Zaragoza, E-50018, Spain

[b]Department of Inorganic Chemistry and Aragón Materials Science Institute (ICMA), University of Zaragoza - C.S.I.C., Pedro Cerbuna 12, Zaragoza, E-50009, Spain

[c]Department of Inorganic Chemistry and Instituto de Síntesis Química y Catalisis Homogenea (ISQCH), University of Zaragoza - C.S.I.C., Pedro Cerbuna 12, Zaragoza, E-50009, Spain

Correspondence email: falvello@unizar.es; milagros@unizar.es

# Supporting information

IMPORTANT: this document contains embedded data - to preserve data integrity, please ensure where possible that the IUCr Word tools (available from http://journals.iucr.org/services/docxtemplate/) are installed when editing this document.

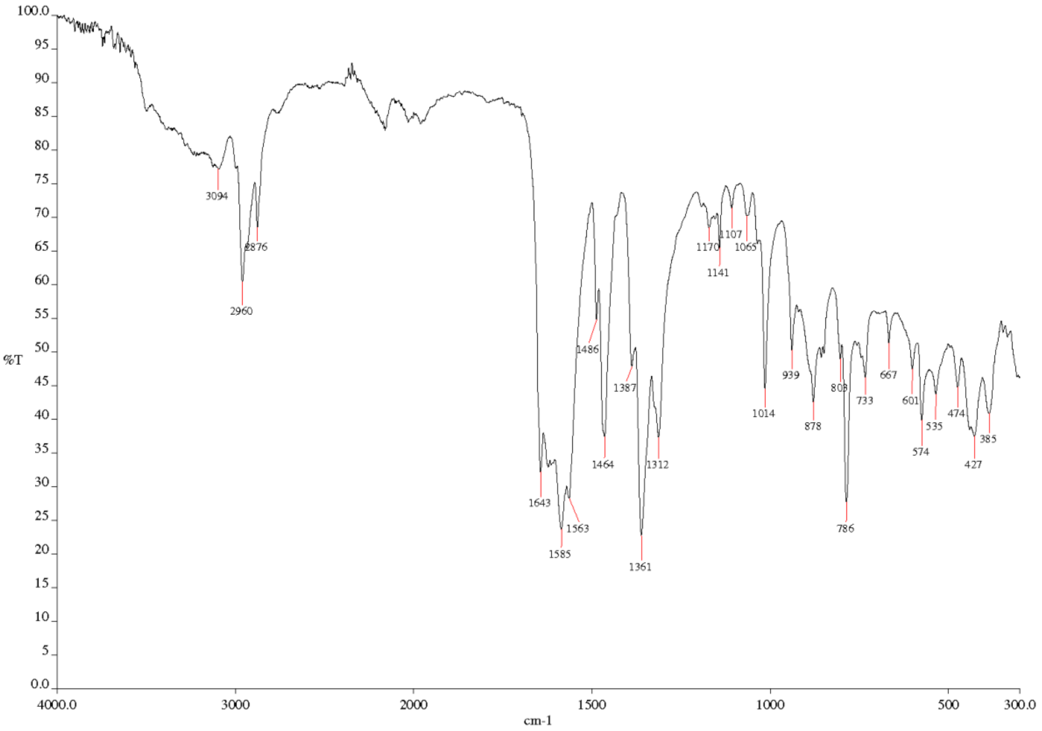


**Figure S1** IR spectrum of ($^{n}$Bu$_4$N)$_2$[*cis*-Co(Or)$_2$(H$_2$O)$_2$]·2H$_2$O, **1**.

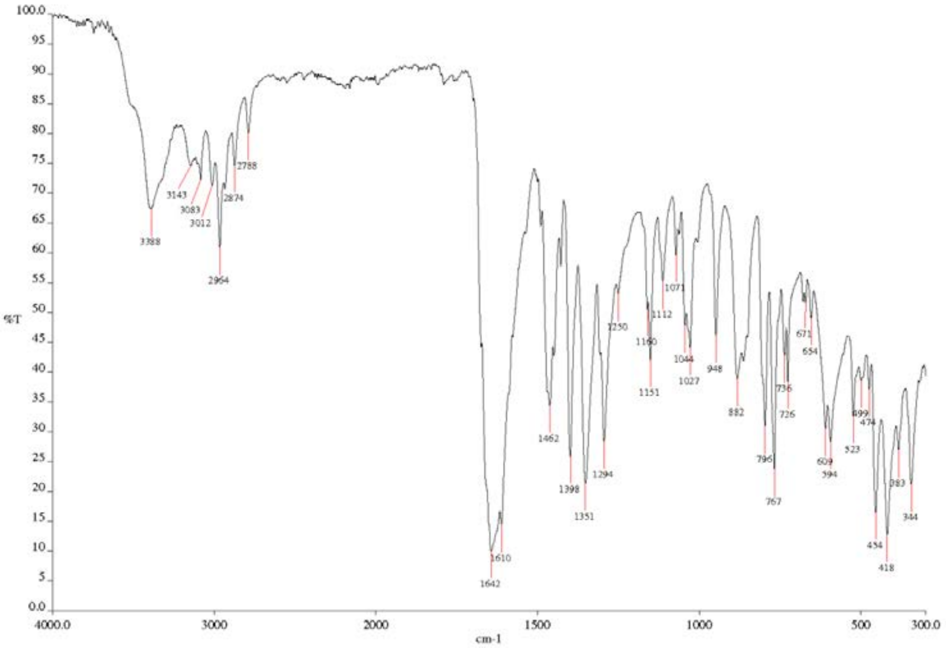


**Figure S2** IR spectrum of ($^{n}$Bu$_4$N)[Co(Or)$_2$(bipy)]·3H$_2$O, **2**.

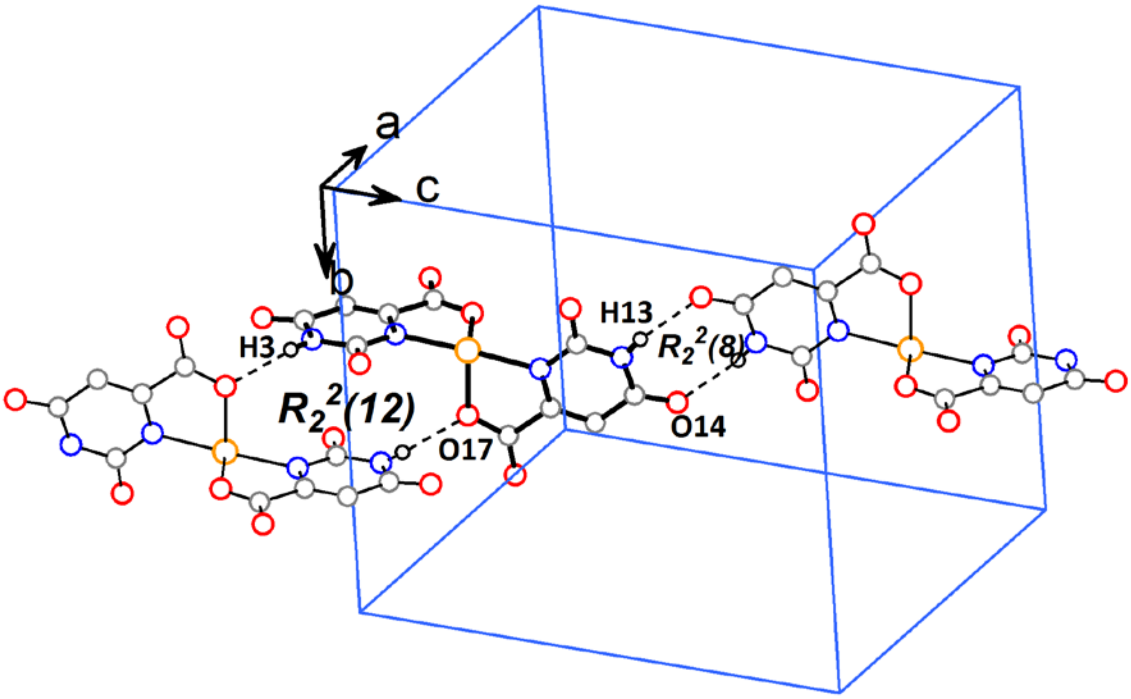


**Figure S3** From the crystal structure of **1**, self-complementary interactions form $R_2^2(12)$ and $R_2^2(8)$ aggregates within a chain of anions propagated along [101].

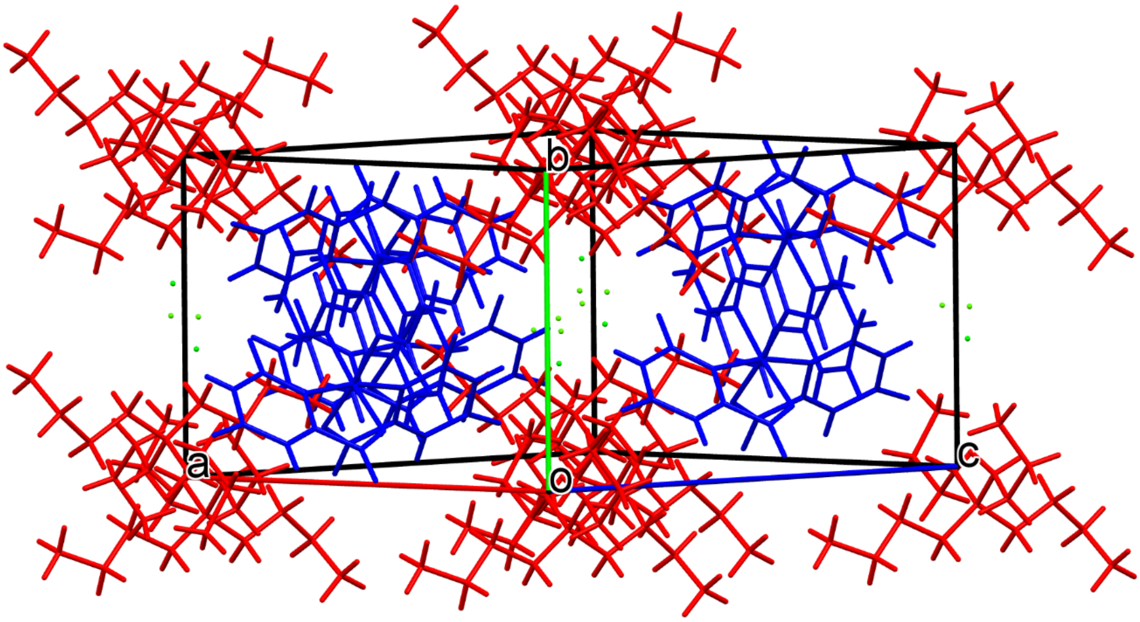


**Figure S4** Packing in the twinned triclinic structure **2b**.
